# Soil fungal and bacterial communities in southern boreal forests of the Greater Khingan Mountains and their relationship with soil properties

**DOI:** 10.1038/s41598-020-79206-0

**Published:** 2020-12-16

**Authors:** Thi-Minh-Dien Vuong, Jian-Yong Zeng, Xiu-Ling Man

**Affiliations:** 1 Department of Soil and Water Conservation and Desertification Control, School of Forestry, Northeast Forestry University, Harbin, 150040 China; 2 Department of International Cooperation, Center of Technology Development and Agricultural Extension, Vietnam Academy of Agricultural Sciences, Hanoi, 100803 Vietnam; 3 Department of Forest Protection, School of Forestry, Northeast Forestry University, Harbin, 150040 China

**Keywords:** Microbial communities, Microbial ecology, Solid Earth sciences

## Abstract

Little is known about the relationship between soil microbial communities and soil properties in southern boreal forests. To further our knowledge about that relationship, we compared the soil samples in southern boreal forests of the Greater Khingan Mountains—the southernmost boreal forest biome in the world. The forests can be divided into boardleaf forests dominated by birch (*Betula platyphylla*) or aspen (*Populus davidiana*) and coniferous forests dominated by larch (*Larix gmelinii*) or pine (*Pinus sylvestris* var. *mongolica*). Results suggested different soil microbial communities and soil properties between these southern boreal forests. Soil protease activity strongly associated with soil fungal communities in broadleaf and coniferous forests (*p* < 0.05), but not with soil bacterial communities (*p* > 0.05). Soil ammonium nitrogen and total phosphorus contents strongly associated with soil fungal and bacterial communities in broadleaf forests (*p* < 0.05), but not in coniferous forests (*p* > 0.05). Soil potassium content demonstrated strong correlations with both soil fungal and bacterial communities in broadleaf and coniferous forests (*p* < 0.05). These results provide evidence for different soil communities and soil properties in southern boreal forest, and further elucidate the explicit correlation between soil microbial communities and soil properties in southern boreal forests.

## Introduction

Soil microbial communities are involved in many important ecological and physiological process in terrestrial ecosystems, such as turnover processes of organic matter, breakdown of pollutants, regulation of mineral nutrient availability, fixation of atmospheric nitrogen (N), and formation of mycorrhiza^1^. In the nutrient cycling in forest ecosystems, the soil microbial communities secrete hydrolases to decompose plant litter and other organic matter^2,3^ and return nutrients back to the soil^3,4^, thus stimulating plant growth^5,6^. In other words, soil microbial communities can shape the soil properties such as nutrient content and hydrolase activity when regulating the microbial degradation process of soil organic matter^1^. Furthermore, soil microbial communities are also regulated by multidimensional soil properties such as soil nutrient content, moisture levels, and pH levels^7^. It has been found that soil pH and moisture shaped the total and active microbial communities in a northern hardwood forest of Michigan, USA^8^. Increasing the soil phosphorus (P) content improved soil microbial respiration^9^ and biomass^10,11^. Other soil nutrients, such as carbon (C) and N, also demonstrated significant effects on soil microbial community structure^12^. These studies documented the two-way interactions between soil microbial communities and soil properties, i.e. soil microbial communities influence soil properties and vice versa^13^. Thus, there have been continued and growing interests to characterize the soil microbial communities and soil properties and elucidate the explicit relationships between them^14–16^.

Boreal forest biomes, also called taiga in Russian, are distinct from tropical, subtropical, and temperate forests^17^, and characterized by a limited number of tree genera such as *Pinus*, *Picea*, *Larix*, *Abies*, *Betula*, and *Populus*^18^. Boreal forests extend across North America and Eurasia, cover about 17 percent the world’s land surface^18^, and play an important role in the global C budget^19^. The Greater Khingan Mountains, which have the best-preserved and largest primeval forest in China, is one of the few locations in China with boreal forests^19^. It is also the southernmost boreal forest biome in the world^20^. Due to the special geography and important ecological functions, boreal forests in the Greater Khingan Mountains have been attached attention from scientific researchers^21^.

In addition, there has been much research on various aspects of boreal forests such as regeneration dynamics^22^, nutrient contents^23^, and status of invasive species^24^. For example, the effect of different factors—i.e., logging^25^, reclamation^26^, wildfire^27^—on boreal forest soil is also a concern. In addition, soil microbial communities in boreal forests have also been reported. *E.g.*, vegetation composition (non-grazed, lichen-dominated, grazed, and bryophyte-dominated sites) determined soil microbial activities in a boreal *Pinus sylvestris* forest of Finland^28^; soil microbial biomass and activity was irrelevant to species composition and diversity of the litter (monocultures or mixtures of tree, dwarf shrub, feather moss) in a boreal forest of northern Sweden as long as plant litter was present on the humus surface^29^. Moreover, previous studies have also recognized the relationship between soil microbial communities and soil properties in boreal forests. For example, linear regressions were observed between soil bacterial abundance and soil pH, total N, and C/N ratio across reclaimed and natural boreal forest in Alberta, Canada^30^. However, relatively little is known about the relationship between soil microbial communities and soil properties in boreal forests. Here, to further our knowledge about that relationship, we want to conduct a research to characterize the soil fungal and bacterial communities of southern boreal forests in the Greater Khingan Mountains, and reveal their relationship with soil properties.

Previous studies have shown that a stable soil microbial community^31–33^ and soil organic C and N stocks^34,35^ are generally formed 30–50 years after afforestation. In other words, soil samples from stands of same age and older than 50 years are better for the present study. However, in the Greater Khingan Mountains Mohe Forest Ecosystem National Positioning Observation and Research Station (hereafter called as Mohe Observation and Research Station), stand ages of boreal forests in the Greater Khingan Mountains are about 20–50 year for broadleaf birch *Betula platyphylla* and aspen *Populus davidiana* forests, and 70–120 years for coniferous larch *Larix gmelinii* and pine *Pinus sylvestris* var. *mongolica* forests. Consequently, we chose to analyze and compare the soil between two broadleaf forests (birch and aspen forests) at an average stand age of 50 years and between two coniferous forests (larch and pine forests) at an average stand age of 96 years respectively.

Before conducting the present study, we hypothesized that soil microbial (i.e., fungal and bacterial) communities were directly associated with soil properties such as total and available nutrient contents, hydrolase activities, moisture, and pH level in southern boreal forests of the Greater Khingan Mountains. When data analysis was completed, this study had further documented the effects of tree species on characteristics of soil microbial communities and soil properties (i.e. birch versus aspen for broad leaf forests, larch versus pine for coniferous forests). It also verified our hypothesis and revealed the relationship between soil microbial communities and soil properties in Chinese southern boreal forests.

## Results

### Soil microbial communities

**Figure 1:**
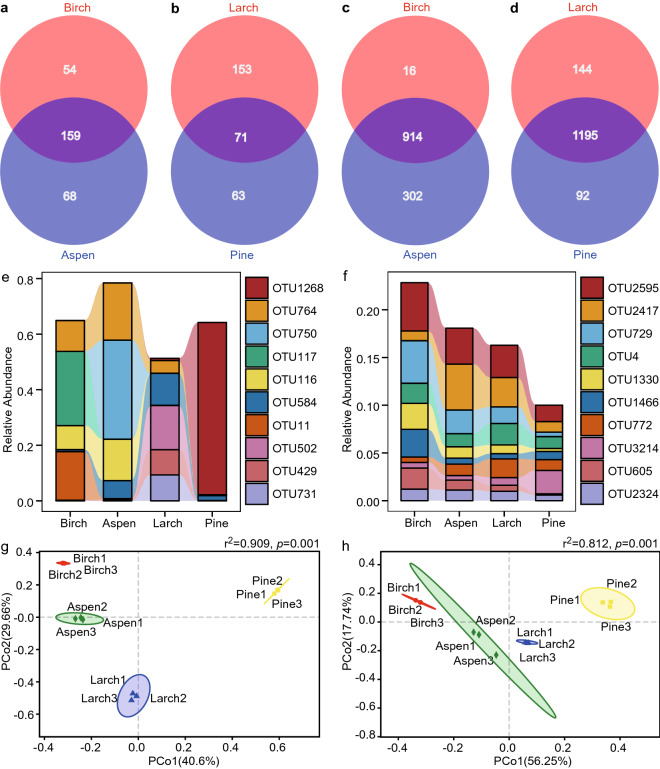
Composition of soil microbial communities at OTU level. Facets a-d are visualizations of Venn analyses: (**a**) soil fungi of birch forest and aspen forest, (**b**) soil fungi of larch forest and pine forest, (**c**) soil bacteria of birch forest and aspen forest, and (**d**) soil bacteria of larch forest and pine forest. Facets **e**–**f** are microbial relative abundance bar charts. Bar charts showing relative abundance of top ten abundant soil fungal (**e**) and bacterial (**f**) OTUs. Facets g and h visualized PCoA results of soil (**g**) fungal and (**h**) bacterial communities. Fiducial limit for confidence ellipses was 0.95. The r^2^ and *p* values at the top right corners are PERMANOVA results.

Soil fungal ITS (ITS1-ITS2 region) sequencing resulted in approximately 805,820 clean reads with an average length of 245 bp, and bacterial 16S rRNA gene (V3–V4 region) sequencing resulted in approximately 641,184 clean reads with an average length of 416 bp (Table S1). Rarefaction curves and core analysis indicated a sufficiently large sample size (Fig. S1). Reads from fungal ITS sequencing were clustered into 386 OTUs, 238 species, 165 genera, 103 families, 58 orders, 28 classes and 9 phyla. Reads from bacterial 16S rRNA gene sequencing were clustered into 1460 OTUs, 674 species, 329 genera, 222 families, 147 orders, 63 classes and 26 phyla. A total of 281 soil fungal OTUs and 1232 soil bacterial OTUs were detected in birch and aspen forests, and 287 soil fungal OTUs and 1431 soil bacterial OTUs were detected in larch and pine forests (Fig. 1).

Among these microbial OTUs detected in birch and aspen forests, 159 (56.58%) fungal OTUs and 914 (74.19%) bacterial OTUs were shared by these two broadleaf forests; but the remaining 122 (43.42%) fungal OTUs and 318 (25.81%) bacterial OTUs specific to birch forest or aspen forest (Fig. 1a,c). Similarly, among these microbial OTUs detected in larch and pine forests, 71 fungal OTUs and 1195 bacterial OTUs were observed in both coniferous forests. The remaining 216 (75.26%) fungal OTUs and 236 (16.49%) bacterial OTUs were particular to larch forest or pine forest (Fig. 1b,d). Consequently, different soil microbial OTUs were observed between birch and aspen forests, and also were observed between larch and pine forests.

Bar charts of community relative abundance revealed OTU117 (*Russula* sp.) to be the dominant fungal OTU in birch forest, OTU750 (*Piloderma* sp.) in aspen forest, OTU502 (*Archaeorhizomyces* sp.) in larch forest, and OTU1268 (*Mortierella elongata*) in pine forest (Fig. 1e). Meanwhile, OTU2595 (*Bradyrhizobium* sp.) was the dominant bacterial OTU in birch and larch forest, OTU2417 (unclassified species in class AD3) in aspen forest, and OTU3214 (unclassified species in order Acidobacteriales) in pine forest (Fig. 1f). To summarize, the difference in dominant fungal and bacterial OTUs were observed not only between birch and aspen forests, but also between larch and pine forests.

All the soil fungal confidence ellipses were discrete in PCoA ordinations (Fig. 1g), and so were the soil bacterial confidence ellipses (Fig. 1h). In addition, PERMANOVA results suggested significant differences between soil microbial communities (*p* = 0.001). This confirmed that differences in beta diversities of soil microbial communities can be observed not only between birch and aspen forests, but also between larch and pine forests (*p* = 0.001) (Fig. 1g,h).

**Figure 2:**
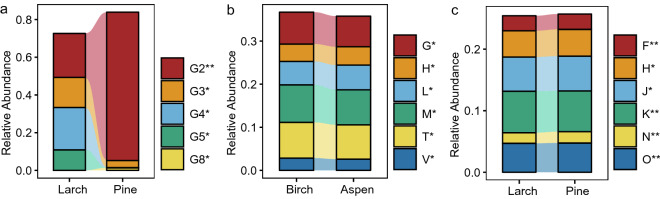
Soil microbial function. Facets a-c shows function guilds (**a**) and categories (**b**, **c**) meet following criteria: with known function; relative abundance > 0.01 in a certain forest; with significant difference relative abundance between some two forests. Asterisks marks the significant difference in relative abundances at levels: * *p* < 0.05; **p < 0.01. Abbreviations of function guilds and categories are showed as follows. G2: Endophyte-Litter Saprotroph-Soil Saprotroph-Undefined Saprotroph; G3: Undefined Saprotroph; G4: Soil Saprotroph; G5: Ectomycorrhizal-Orchid Mycorrhizal-Root Associated Biotroph; G8: Arbuscular Mycorrhizal; F: Nucleotide transport and metabolism; G: Carbohydrate transport and metabolism; H: Coenzyme transport and metabolism; J: Translation, ribosomal structure and biogenesis; K: Transcription; L: Replication, recombination and repair; M: Cell wall/membrane/envelope biogenesis; N: Cell motility; O: Posttranslational modification, protein turnover, chaperones; T: Signal transduction mechanisms; V: Defense mechanisms.

PERMANOVA results indicated that overall differences in functional fungal composition were significant between forests (i.e. birch versus aspen, larch versus pine, *p* = 0.001). Statistical results showed that no significant difference (*p* > 0.05) was observed in the relative abundance of any of the fungal functional guilds between birch and aspen forests. However, the relative abundances of five fungal functional guilds significantly differed (*p* < 0.05) between larch and pine forests. Function guild Endophyte-Litter Saprotroph-Soil Saprotroph-Undefined Saprotroph (guild G2) was the most abundant fungal function guild with significantly different relative abundances between larch and aspen forest (*p* < 0.05). However, the relative abundance of guild G2 in pine forest (78.68%) was 3.38 times to that in larch forest (23.27%). Function guilds Soil Saprotroph (guild G4) and Ectomycorrhizal-Orchid Mycorrhizal-Root Associated Biotroph (guild G5) maintained relative abundances higher than one-tenth in larch forest (i.e. 22.50% and 10.87%, respectively), but lower than one percent in pine forest. Similarly, relative abundance of function guild Undefined Saprotroph (guild G3) was 15.97% in larch forest and only 3.74% in pine forest. Relative abundance of guild Arbuscular Mycorrhizal (guild G8) was 0.01% in larch forest, but 13.8% in pine forest (Fig. 2a).

PERMANOVA results also suggest significant overall differences in functional bacterial composition between forests (i.e. birch versus aspen, larch versus pine, *p* = 0.001). Statistical results also suggested six bacterial function categories maintaining significant difference in relative abundances between birch and aspen forests (*p* < 0.05) (Fig. 2b). Consistently, there were six function categories that had different relative abundances between larch and pine forests (*p* < 0.05) (Fig. 2c). However, coenzyme transport and metabolism function category (category H) was the only category maintaining different relative abundance not only between broadleaf forests but also between coniferous forests (i.e. birch VS aspen forest, and larch VS pine forest). Moreover, differing from fungal function guilds, the maximum difference in relative abundance values of bacterial function categories were only 0.33% (Fig. 2a–c).

### Soil properties

**Figure 3:**
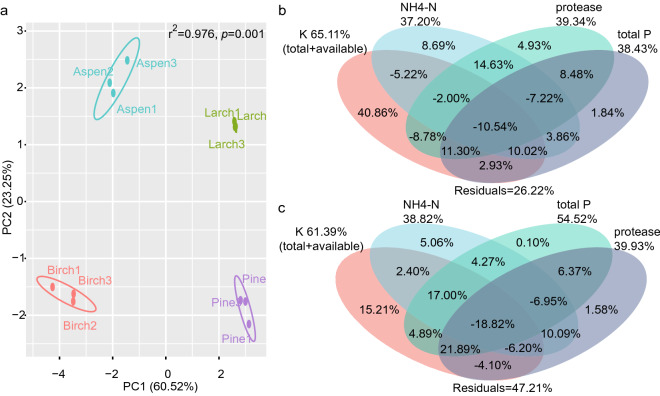
Soil properties. Facet a visualized the PCA results. Fiducial limit for confidence ellipses in this PCA visualization was 0.95. The r^2^ and *p* values at the top right corner are PERMANOVA results. Facets b and c were visualizations of variance partitioning analysis (VPA). Soil properties that highlighted in RDA/CCA analysis were employed to performed VPA with soil fungal (**b**) and bacterial (**c**) communities of these four southern boreal forests respectively.

PCA results for soil properties showed that the first and second axes explained 83.77% of the variance in total, and the visualization revealed discrete grouping ellipses. Furthermore, PERMANOVA results suggested the significantly different soil properties between southern boreal forests in the Greater Khingan Mountains (*p* = 0.001) (Fig. 3a). These results suggest the significantly different soil properties not only between birch and aspen forests, but also between larch and pine forests (Table 1). Soil total organic C and total N contents in birch forest were significantly higher than that in aspen forest (*p* < 0.05). Soil total and available P content, available K content, protease activity, and pH in birch forest were significantly lower than that in aspen forest (*p* < 0.05). Soil pH in larch forest was significantly higher than that in pine forest, however, soil properties including NH_4_-N, NO_3_-N, dissolved organic C, total and available P, total and available K, and protease showed higher content or activity in pine forest rather than larch forest (*p* < 0.05) (Table 1). But soil urease activity, sucrase activity, and moisture did not show any significant difference (*p* > 0.05) between birch and aspen forests or between larch and pine forests (Table S2).

**Table 1:** Soil properties in southern boreal forests in Greater Khingan Mountains.

Soil properties	Birch	Aspen	Larch	Pine	Birch-Aspen	Larch- Pine
NH_4_-N	20.28 ± 0.49	19.45 ± 0.63	26.33 ± 1.68	31.41 ± 0.63	NS	**
NO_3_-N	1.51 ± 0.03	1.51 ± 0.07	1.53 ± 0.03	2.21 ± 0.21	NS	**
Dissolved organic C	99.66 ± 8.92	87.52 ± 5.20	108.02 ± 10.47	182.36 ± 9.27	NS	**
Available P	27.98 ± 0.88	31.50 ± 0.75	18.20 ± 0.25	22.87 ± 0.71	**	**
Available K	103.05 ± 5.16	289.79 ± 14.99	256.47 ± 24.27	221.37 ± 12.63	**	NS
Total organic C	47.40 ± 2.51	39.57 ± 2.01	83.84 ± 0.90	88.54 ± 6.02	*	NS
Total N	3.35 ± 0.13	2.88 ± 0.12	4.32 ± 0.29	4.60 ± 0.02	*	NS
Total P	1.47 ± 0.10	2.34 ± 0.37	1.44 ± 0.08	2.08 ± 0.03	*	**
Total K	18.05 ± 0.71	19.63 ± 1.01	17.48 ± 0.41	18.93 ± 0.27	NS	**
Protease	0.40 ± 0.05	0.58 ± 0.04	0.51 ± 0.01	0.67 ± 0.01	**	**
Cellulose	0.41 ± 0.02	0.44 ± 0.06	0.42 ± 0.03	0.65 ± 0.01	NS	**
pH	5.57 ± 0.02	5.88 ± 0.01	4.84 ± 0.01	4.50 ± 0.02	**	**

Ammonium nitrogen (NH_4_-N); nitrate nitrogen (NO_3_-N); C, N, P, and K are common abbreviations of elements. Units are g/kg for contents of total organic C, total N, total P, and total K; while mg/kg for other nutrient contents. Meanings of hydrolase activities were showed in “Methods” Section. Moistures are presented as percentages. The last two columns indicated the statistical significance between boreal forests: NS (*p* > 0.05); * (*p* < 0.05); ** (*p* < 0.01). Soil properties with no significant difference between boreal forests were omitted here, but they are showed in Table S3.

### Relationship between soil microbial community and soil properties

RDA/CCA suggested that strong association was observed between soil microbial communities and soil properties in these southern boreal forests (Table 2). Soil fungal communities in these two broadleaf forests were strongly associated with contents of soil NH_4_-N (r^2^ = 0.892, *p* = 0.038), total P (r^2^ = 0.975, *p* = 0.044), and total potassium (K) (r^2^ = 0.961, *p* = 0.006), and protease activity (r^2^ = 0.990, *p* = 0.039). Soil fungal communities in the two coniferous forests were strongly associated with soil available K content (r^2^ = 0.993, *p* = 0.038), total organic C content (r^2^ = 0.926, *p* = 0.007), pH level (r^2^ = 0.998, *p* = 0.022), and protease activity (r^2^ = 0.892, *p* = 0.036). Soil bacterial communities in these two broadleaf forests were strongly associated with soil NH_4_-N content (r^2^ = 0.951, *p* = 0.007), dissolved organic C content (r^2^ = 0.849, *p* = 0.031), available K content (r^2^ = 0.900, *p* = 0.036), total N content (r^2^ = 0.858, *p* = 0.039), total P content (r^2^ = 0.876, *p* = 0.003), pH level (r^2^ = 0.793, *p* = 0.033), and cellulose activity (r^2^ = 0.822, *p* = 0.022). Soil bacterial communities in coniferous forests were strongly associated with soil available K content (r^2^ = 0.995, *p* = 0.018). However, soil properties including NO_3_-N, available P, urease, sucrase, and moisture did not show any strong association (*p* > 0.05) with soil microbial communities in these broadleaf or coniferous forests (Table S4).

**Table 2:** Relationship between soil microbial communities and soil properties.

Soil properties	Soil fungi in broadleaf forests		Soil fungi in coniferous forests		Soil bacteria in broadleaf forests		Soil bacteria in coniferous forests	
	r^2^	*p* values	r^2^	*p* values	r^2^	*p* values	r^2^	*p* values
NH_4_-N	0.892	0.038*	0.599	0.282	0.951	0.007*	0.709	0.113
Dissolved organic C	0.932	0.081	0.840	0.056	0.849	0.031*	0.520	0.368
Available K	0.731	0.150	0.993	0.038*	0.900	0.036*	0.995	0.018*
Total organic C	0.309	0.564	0.926	0.007*	0.333	0.519	0.861	0.076
Total P	0.975	0.044*	0.802	0.128	0.876	0.003*	0.833	0.094
Total K	0.961	0.006*	0.649	0.208	0.820	0.076	0.723	0.182
pH	0.99	0.056	0.998	0.022*	0.793	0.033*	0.990	0.086
Protease	0.99	0.039*	0.892	0.036*	0.756	0.103	0.896	0.069
Cellulose	0.951	0.100	0.179	0.822	0.822	0.022*	0.333	0.511

Ammonium nitrogen (NH_4_-N); nitrate nitrogen (NO_3_-N); C, N, P, and K are common abbreviations of elements. Asterisks marked the significant statistical difference (*p* < 0.05). Variance partitioning values of ordination axes were omitted here, and so did soil properties that are not strongly associated with soil microbial communities, but they are showed in Table S4.

In summary, several interesting associations can be found as follows: soil NH_4_-N and total P contents were strongly associated with both soil fungal and bacterial communities in boreal broadleaf forests (*p* < 0.05). Soil protease activity strongly correlated with soil fungal communities in boreal broadleaf and coniferous forests (*p* < 0.05). Soil available K contents were strongly associated with soil fungal communities in boreal coniferous forests and soil bacterial communities in both boreal broadleaf and coniferous forests (*p* < 0.05). In addition, although strong association was not observed between soil available K contents and soil fungal communities in boreal broadleaf forests (*p* > 0.05), it showed between soil total K contents and soil fungal communities (*p* < 0.05). In other words, soil fungal and bacterial communities always maintained significant associations with soil total/available K (Table 2).

Furthermore, the contribution of these highlighted soil properties—i.e., NH_4_-N, total P, protease, and total and available K—to soil fungal and bacterial communities in these four boreal forests were quantified by variance partitioning analysis (VPA) (Fig. 3b,c). Results showed that the variances of soil fungal communities could be explained by K nutrition of 65.11%, NH_4_-N contents of 37.20%, total P contents of 38.34%, and protease activities of 39.34% (Fig. 3b). Variances of soil bacterial communities could be explained by K nutrition of 61.39%, NH_4_-N contents of 38.82%, total P contents of 54.52%, and protease activities of 39.93% (Fig. 3c). While the total explanatory ratio of that these soil properties was 73.78% for soil fungal communities and 52.79% for bacterial communities (Fig. 3b,c).

## Discussion

The present study analyzed the differences in soil microbial communities and soil properties between southern boreal forests of the Greater Khingan Mountains, China. Different composition, diversity, and function of these southern boreal forests were documented here. Results also showed that total and available P contents, protease activities, and pH levels were significantly different between southern boreal broadleaf forests and between southern boreal coniferous forests. In short, the present study demonstrates difference in soil microbial communities and soil properties between southern boreal forests in the Greater Khingan Mountains, China. It is consistent with the earlier findings^36,37^, and provide new evidence for the effects of tree species on soil communities and soil properties in southern boreal forest. The previous study documented that tree species showed a positive effect on soil C and N stocks in an Iranian temperate forest^38^. Microbial C and N and their ratio to total soil organic C or total N were also documented to be significantly affected by tree species in southern boreal forests of Canada^39^. It has been also reported that soil bacterial community structure and function in German temperate deciduous forests were governed by the tree species^40^. Soil fungal communities also varied between Chinese subtropical evergreen and deciduous forests^37^. Those previous studies have confirmed that tree species contribute to differences in soil microbial communities and soil properties^41^.

Soil protease activity was also the only detected soil property that consistently maintained strong association with soil fungal communities in these southern boreal forests, but not maintained any strong association with soil bacterial communities. Consistent with the previous studies^42,43^, this indicated that soil properties could be correlated differently with the soil fungal and bacterial communities in these southern boreal forests. Furthermore, it has been reported that, soil protease activity is a limit to the first step of soil N mineralization^44^. In other words, soil protease released by soil fungi—the major groups of saprotrophs—may explain the different decomposition pattern of plant litter in southern boreal forests^44^.

Results also suggested soil NH_4_-N and total P content strongly associated with soil fungal and bacterial communities in broadleaf forests, but not in coniferous forests. This suggested that the relationship between soil microbial communities and soil properties can be different between boreal broadleaf and coniferous forests, which is consist with previous observations in temperate^45^ and subtropical forests^46^. Another interesting result is that, soil fungal and bacterial communities in these southern boreal broadleaf and coniferous forests are significantly associated with soil total or available K content. Moreover, soil K content is critical for pest and disease resistance in plants^47^, and determines the crop productivity and quality^48^. Thus, K content should be concerned in soil and forest management in these southern boreal forests^49,50^. This finding is in contrast to subtropical forests where K is irrelevant to soil microbial activity^51^. The contrasting findings might be due to the differences between studies in sampling depth^52^, forest type^53^, and latitude^54^. However, limited comparable data makes it still become a question that whether the present relationship between soil microbial communities and soil properties in these Chinese southern boreal forests is generally apply to other boreal forests.

According to previous studies, plant litter which is a critical nutrient input source for forest soil^55^ can shape different soil microbial communities^53^. Moreover, two-way interaction has been also reported between soil microbial communities and soil properties in previous study^13^. Thus, it is believed that the litter composition is critical for the differences in soil microbial communities and soil properties between these southern boreal forests^56^. Additionally, this inference droved us to further study the effects litter composition on soil-microbiota-litter microecosystem.

## Conclusion

This study documented differences in soil microbial communities and soil properties between southern boreal broadleaf/coniferous forests in the Greater Khingan Mountains, China. It also revealed several interesting relationship between soil microbial communities and soil properties in Chinese southern boreal forests: (a) soil protease activity strongly correlated with soil fungal communities in Chinese southern boreal broadleaf and coniferous forests (*p* < 0.05), but not with soil bacterial communities (*p* > 0.05); (b) soil NH_4_-N and total P content strongly correlated with soil fungal and bacterial communities in southern boreal broadleaf forests (*p* < 0.05), but not in coniferous forests (*p* > 0.05); (c) soil K content demonstrated strong correlations with both soil fungal and bacterial communities in southern boreal broadleaf and coniferous forests (*p* < 0.05). Overall, this study further documented the effects of tree species on characteristics of soil microbial communities and soil properties of southern boreal broadleaf and coniferous forests; it also furthered our understanding about relationship between soil microbial communities and soil properties in Chinese southern boreal forests. However, limited comparable data makes it still become a question that whether the present relationship is generally apply to other boreal forests.

## Methods

### Study site information

The study was performed in the Mohe Observation and Research Station (53° 17′–53° 30′ N, 122° 06′–122° 27′ E) in the Greater Khingan Mountains of northeast China. Based on the Mohe Observation and Research Station, the mean annual temperature of the study site was − 5.5 °C and the mean annual precipitation was 425 mm. Rainfall was concentrated from July to August. The frost period generally lasted from late October to early May of the following year. Cool temperature and long frost period led to permafrost in the study site. Four natural forests that dominated by single species there (i.e., one of birch, aspen, larch, and pine) were selected for soil sample collection. The canopy density in these sample forests was 0.8. The stand age was 96 ± 5 years for larch forest, 96 ± 7 years for pine forest, and 50 ± 5 years for both birch and aspen forests. Considering the difference in stand age might generate false positives, we only analyzed and compared birch forest with aspen forest (broadleaf forests) and larch forest with pine forest (coniferous forests).

### Soil sampling and management

Three sampling areas were randomly selected in each forest as replicates. Topsoil cylinders (17 cm in diameter and 5 cm in height) was collected via the five diagonal point sampling method^57^ after litter removal from each sampling area (20 m × 30 m). Soil samples were sealed in bags, stored on ice in an insulated box, and transported to the lab. In the lab, soil samples were sieved using 2 mm mesh to remove impurities such as leaves, roots, and stones. Equal amounts of clean soil samples from five sampling point of each sampling area were mixed as soil samples for analyses of soil microbial community and soil properties. Fresh soil samples with a particle size ≤ 2 mm were used to analyze soil microbial community, dissolved organic C content, NH_4_-N content, NO_3_-N content, and soil moisture. Air-dried soil samples with a particle size ≤ 2 mm were used to analyze the available K content, available P content, pH level, and enzyme activities. Air-dried soil samples with a particle size ≤ 0.149 mm were used to analyze the soil total nutrient contents including total organic C, total N, total K, and total P.

### Soil microbial gene sequencing

Microbial DNA was extracted from the soil samples using FastDNA SPIN Kit for Soil (MP Biomedicals United States, USA) following the manufacturer’s instructions. DNA quality was checked using a NanoDrop 2000 UV–Vis spectrophotometer (Thermo Scientific, USA) and 2% agarose gel. We used the primers 338F and 806R to amplify soil bacterial 16S rRNA gene (V3–V4 region) and ITS1F and ITS2R to amplify the soil fungal internal transcribed spacer (ITS; ITS1–ITS2 region) (Table S2) for sequencing^58^. The target was amplified with TransStart Fastpfu DNA Polymerase (TransGen Biotech, China), purified with AxyPrep DNA Gel Extraction Kit (Axygen Biosciences, USA), and quantified using QuantiFluor-ST (Promega, USA) as per the manufacturers’ instructions. The purified DNA was sequenced using TruSeq DNA Sample Prep Kit (Illumina, USA) following the manufacturer’s protocol.

### Soil chemical property analysis

AutoAnalyzer 3 high-resolution digital colorimeter (DKSH, China) was used to analyze the soil total N, NH_4_-N, and NO_3_-N contents^59^. Soil total and dissolved organic C contents were determined using Vario TOC cube elemental analyzer (DKSH, China)^60^. Soil total and available K contents were determined using flame spectrophotometer^61^. Soil total and available P contents were determined by spectrophotometry^62^. Colorimetric analyses were also conducted to measure the activities of soil urease^63^, protease^64^, sucrase^65^, and cellulase^66^. In addition, urease activity is expressed as the amount of NH_4_^+^ produced per gram of soil per day (mg·g^−1^·d^−1^); protease activity is expressed as the amount of amino acid produced per gram of soil per day (mg·g^−1^·d^−1^); sucrase activity is expressed as the amount of reducing sugar produced per gram of soil per hour (mg·g^−1^·h^−1^); and cellulase activity is expressed as the amount of glucose produced (μg) per gram of soil per hour (μg·g^−1^·h^−1^). Soil pH and moisture were determined following the methods described in previous studies^8^.

### Data preparation and analysis

Isanger Cloud Platform (https://www.i-sanger.com), which is provided by Majorbio Co., Ltd. (Shanghai, China), is an integration of common bioinformatic tools, such as Muthor, FUNGuild, PICRUSt, CRAN R, LEfSe, and ggplot2. Bioinformatic analyses of soil microbial communities were done using Isanger Cloud Platform v4.0. Raw sequences of 16S rRNA gene and ITS were demultiplexed and quality-filtered using Trimmomatic version 0.39^67^ and merged using FLASH version 1.2.11^68^ with the following criteria: (a) Reads were truncated at any site receiving an average quality score < 20 over a 50 bp sliding window; (b) Primers with two nucleotide mismatches were allowed, and reads containing ambiguous bases were removed; and (c) Sequences with overlaps longer than 10 bp were merged according to their overlap sequence. RDP classifier version 11.5 (Bayesian algorithm) was used to classify the operational taxonomic units (OTU) with 97% sequence similarity^69^. Additionally, the data set was also subjected to quantity-filtering and normalization as follows: (a) Reads with abundance lower than five in three replicates were removed from the data set. (b) Sequence numbers in each replicate were normalized by randomly selecting the minimum sequence number in replicates.

Rarefaction analysis was carried out at the OTU level with Mothur version 1.30.2 to assess the sequencing depth^70^. Venn analysis was performed at the OTU level using VennDiagram package to study the microbial identity composition^71^. Bar chart of relative abundance at the OTU level was plotted with ggplot2 to study the microbial relative abundance in each microbial community^72^. Microbial β-diversity was assessed at OTU level with principal coordinates analysis (PCoA, Bray–Curtis distance algorithm) and PERMANOVA analysis (Bray–Curtis distance, permutations = 999) using R package vegan^73^, and visualized with ggplot2^72^.

Statistical differences in soil properties were assessed using Student’s *t*-test^74^, principal component analysis (PCA, prcomp R function)^75^ and PERMANOVA analysis (Bray–Curtis distance, permutations = 999)^73^. PCA ordinations and PERMANOVA results visualized with ggbiplot R package^76^. Soil properties were employed as environmental factors in redundancy analysis (RDA) and canonical correspondence analysis (CCA) to analyze the relationship between soil microbial communities and soil properties, and the RDA and CCA were performed using vegan R package and the results were tested with R function anova^73^. In addition, no more than five soil properties can be included in each RDA/CCA with microbial communities at a time owing to limitations of the algorithm and sample size. Consequently, soil properties were divided into three groups to perform RDA/CCA, respectively, as follows: (a) contents of dissolved organic C, NH_4_-N, NO_3_-N, available K, and available P; (b) contents of total organic C, total N, total K, and total P, and pH level; (c) activities of urease, protease, sucrase, and cellulase, and moisture (consistent results in the preliminary analysis indicated this strategy is feasible). Contributions of soil properties to differences in soil fungal and bacterial communities among these four boreal forests were quantified by variance partitioning analysis (VPA)^73^. In addition, functional classification of soil microbial communities was performed using FUNGuild version 1.0 tool (ITS)^77^ or PICRUSt version 1.1.0 tool (16S rRNA gene)^78^. PERMANOVA analysis (Bray–Curtis distance, permutations = 999) was carried out to evaluate the overall differences of known fungal function guilds and bacterial function categories between forests^73^ . Student’s *t*-test was carried out to evaluate the statistical significance of each fungal function guilds and bacterial function categories between soil samples^74^. Function guilds/categories meet following criteria were visualized with ggplot2^72^: with known function; relative abundance > 0.01 in a certain forest; with significantly different relative abundance between two forests.

## Supplementary information

Supplementary Information.

## Data Availability

Sequence data supporting the findings of this study have been deposited at NCBI under the BioProject number PRJNA624797.
