# Supplementary material for: Soil fungal and bacterial communities in southern boreal forests of the Greater Khingan Mountains and their relationship with soil properties
Source: Sci Rep. 2020 Dec 16;10:22025. doi: 10.1038/s41598-020-79206-0 (PMC7745012; doi:10.1038/s41598-020-79206-0)
Supplement: Supplementary file 1 — Supplementary Information. [file 41598_2020_79206_MOESM1_ESM.pdf]

Soil Fungal and Bacterial Communities in Southern Boreal Forests of the Greater Khingan Mountains and their Relationship with Soil Properties

Thi-Minh-Dien Vuong[1,2 #], Jian-Yong Zeng[3, #], Xiu-Ling Man[1, *]

[#] Thi-Minh-Dien Vuong and Jian-Yong Zeng contributed equally to this work

[*] Xiu-Ling Man is the corresponding author

[1]Department of Soil and Water Conservation and Desertification Control, School of Forestry, Northeast Forestry University, Harbin, China, 150040

[2] Department of International Cooperation, Center of Technology Development and Agricultural Extension, Vietnam Academy of Agricultural Sciences, Hanoi, Vietnam, 100803

[3] Department of Forest Protection, School of Forestry, Northeast Forestry University, Harbin, China,150040

*** Correspondence:**

Xiu-Ling, Man; mannefu@126.com

Supplementary materials

Supplementary table

Table S1 Sequence information

Gene	Region	Samples	Clean reads	Bases (bp)	length (bp)
ITS gene	ITS1-ITS2	12	805820	197088266	245
16S rDNA	V3–V4	12	641184	266799031	416

Table S2 Primers in the present study.

Primer name	Sequence (5’→3’)	Usage
338F	ACTCCTACGGGAGGCAGCAG	16S amplification
806R	GGACTACHVGGGTWTCTAAT	
ITS1F	CTTGGTCATTTAGAGGAAGTAA	ITS amplification
ITS2R	GCTGCGTTCTTCATCGATGC	

Table S3 Soil properties in southern boreal forests in Greater Khingan Mountains (full edition)

Soil properties	Birch	Aspen	Larch	Pine	Birch VS Aspen	Larch VS Pine
NH4-N	20.28 ± 0.49	19.45 ± 0.63	26.33 ± 1.68	31.41 ± 0.63	NS	**
NO3-N	1.51 ± 0.03	1.51 ± 0.07	1.53 ± 0.03	2.21 ± 0.21	NS	**
Dissolved organic C	99.66 ± 8.92	87.52 ± 5.20	108.02 ± 10.47	182.36 ± 9.27	NS	**
Available P	27.98 ± 0.88	31.50 ± 0.75	18.20 ± 0.25	22.87 ± 0.71	**	**
Available K	103.05 ± 5.16	289.79 ± 14.99	256.47 ± 24.27	221.37 ± 12.63	**	NS
Total organic C	47.40 ± 2.51	39.57 ± 2.01	83.84 ± 0.90	88.54 ± 6.02	*	NS
Total N	3.35 ± 0.13	2.88 ± 0.12	4.32 ± 0.29	4.60 ± 0.02	*	NS
Total P	1.47 ± 0.10	2.34 ± 0.37	1.44 ± 0.08	2.08 ± 0.03	*	**
Total K	18.05 ± 0.71	19.63 ± 1.01	17.48 ± 0.41	18.93 ± 0.27	NS	**
Urease	5.54 ± 0.06	5.48 ± 0.09	6.18 ± 0.06	5.88 ± 0.27	NS	NS
Protease	0.40 ± 0.05	0.58 ± 0.04	0.51 ± 0.01	0.67 ± 0.01	**	**
Sucrase	0.34 ± 0.07	0.36 ± 0.04	0.62 ± 0.02	0.64 ± 0.01	NS	NS
Cellulose	0.41 ± 0.02	0.44 ± 0.06	0.42 ± 0.03	0.65 ± 0.01	NS	**
Moisture	29.46 ± 0.58	30.26 ± 0.13	50.16 ± 0.19	49.95 ± 0.05	NS	NS
pH	5.57 ± 0.02	5.88 ± 0.01	4.84 ± 0.01	4.50 ± 0.02	**	**

Abbreviations: ammonium nitrogen (NH_4-N); nitrate nitrogen (NO_3-N); C, N, P, and K are common abbreviations of elements. Units are g/kg for contents of total organic C, total N, total P, and total K; while mg/kg for other nutrient contents. Meanings of hydrolase activities were showed in section Methods. Moistures are presented as percentages. The last two columns indicated the statistical significance between boreal forests: NS (p > 0.05); * (p <0.05); ** (p <0.01).

Table S4 Relationship between soil microbial communities and soil properties (full edition)

Soil properties	Soil fungi in broadleaf forests				Soil fungi in coniferous forests				Soil bacteria in broadleaf forests				Soil bacteria in coniferous forests			
	RDA1	RDA2	r^2	*p* values	CCA1	CCA2	r^2	*p* values	RDA1	RDA2	r^2	*p* values	RDA1	RDA2	r^2	*p* values
NH_4-N	-0.955	0.296	0.892	0.038 *	-0.863	-0.505	0.599	0.282	-0.993	-0.119	0.951	0.007 *	-0.739	-0.674	0.709	0.113
NO_3-N	-1.000	0.030	0.878	0.075	-0.030	1.000	0.118	0.803	-1.000	0.012	0.672	0.190	-0.991	0.136	0.001	0.997
Dissolved organic C	-0.996	0.091	0.932	0.081	-0.758	-0.653	0.840	0.056	-0.985	-0.174	0.849	0.031 *	-0.911	-0.412	0.520	0.368
Available P	-1.000	0.014	0.966	0.050	0.999	0.045	0.883	0.078	-0.901	-0.433	0.792	0.064	0.965	0.262	0.883	0.119
Available K	0.910	-0.415	0.731	0.150	1.000	-0.002	0.993	0.038 *	0.570	0.822	0.900	0.036 *	0.982	0.189	0.995	0.018 *
Total organic C	-0.993	-0.118	0.309	0.564	-0.948	0.318	0.926	0.007 *	-0.880	0.475	0.333	0.519	-1.000	0.027	0.861	0.076
Total N	-0.664	0.748	0.843	0.089	-1.000	0.033	0.837	0.094	-0.992	-0.126	0.858	0.039	-0.963	-0.268	0.843	0.132
Total P	-0.991	0.135	0.975	0.044 *	0.991	0.135	0.802	0.128	-0.956	-0.293	0.876	0.003 *	0.990	-0.142	0.833	0.094
Total K	-0.953	0.302	0.961	0.006 *	0.931	-0.365	0.649	0.208	-0.921	-0.391	0.820	0.076	0.946	-0.326	0.723	0.182
pH	1.000	-0.026	0.990	0.056	0.996	0.089	0.998	0.022 *	0.934	0.358	0.793	0.033 *	0.991	0.132	0.990	0.086
Urease	0.990	-0.142	0.453	0.425	-0.677	-0.736	0.443	0.358	0.686	0.728	0.820	0.113	-0.413	-0.911	0.803	0.069
Protease	-1.000	-0.029	0.990	0.039 *	0.973	0.229	0.892	0.036 *	-0.929	-0.371	0.756	0.103	0.943	0.333	0.896	0.069
Sucrase	-0.518	0.856	0.564	0.242	0.910	0.414	0.076	0.914	-0.998	-0.069	0.593	0.233	0.426	0.905	0.241	0.631
Cellulose	-1.000	0.011	0.951	0.100	0.987	-0.164	0.179	0.822	-0.976	-0.219	0.822	0.022 *	0.582	0.813	0.333	0.511
Moisture	0.769	-0.639	0.838	0.083	0.997	0.073	0.565	0.296	0.797	0.604	0.530	0.294	0.994	0.108	0.557	0.332

Abbreviations: ammonium nitrogen (NH_4-N); nitrate nitrogen (NO_3-N); C, N, P, and K are common abbreviations of elements. Asterisks marked the significant statistical difference (p <0.05).

Supplementary figures

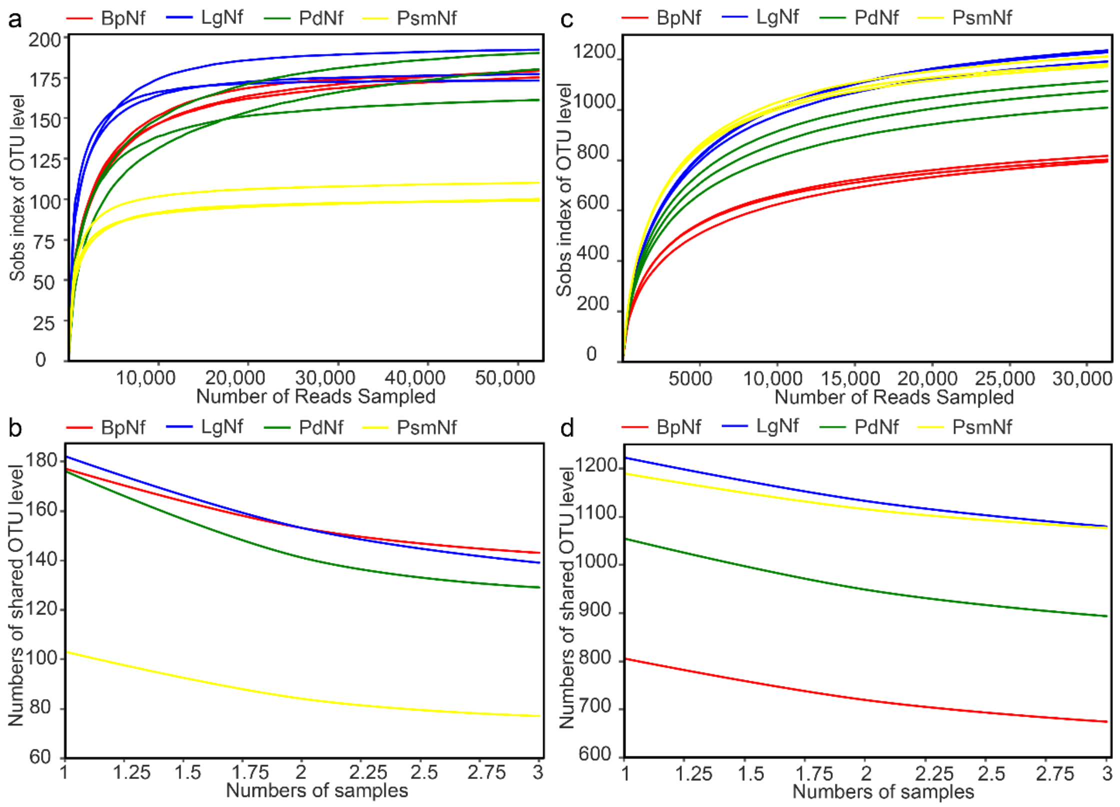

Figure S1 Adequacy analysis of sample size at the operational taxonomic unit level. (a) Rarefaction cure analysis for soil fungi; (b) Core analysis for soil fungi; (c) Rarefaction curve analysis for soil bacteria; (d) Core analysis for soil bacteria.
